# A Cohort Study Comparing Pediatric Patients with Overweight and Obesity in the Military Health System

**DOI:** 10.1089/chi.2021.0040

**Published:** 2021-09-21

**Authors:** Dimas C. Espinola, Cara Olsen, Amanda Banaag, Tracey Pérez Koehlmoos

**Affiliations:** ^1^ Department of Preventive Medicine and Biostatistics, Uniformed Services University of Health Sciences, Bethesda, MD, USA.; ^2^ Department of Pediatrics, Pediatric Subspecialty Clinic, Brooke Army Medical Center, Houston, TX, USA.; ^3^ The Henry M. Jackson Foundation for the Advancement of Military Medicine, Inc., Bethesda, MD, USA.

**Keywords:** child welfare, Military Health System, national security, pediatric health, pediatric overweight

## Abstract

***Background:*** National Health and Nutrition Examination Survey data from the 1960s to 2010s confirm that pediatric obesity rates are increasing. To assess obesity in the Military Health System (MHS), we evaluated a pediatric cohort's trends in BMI categorization from 2009 to 2016.

***Methods:*** We identified two age-based pediatric cohorts in the United States using the MHS Data Repository. We tracked them for BMI from 2009 to 2016. We calculated BMI percentiles and *z*-scores using validated growth charts, and biologically implausible BMI *z*-scores were removed from analyses. Using the Stuart-Maxwell test, we assessed the percent change in BMI categorization from 2009 to 2016 and stratified by age group.

***Results:*** Our cohort consisted of 130,675 pediatric patients (52.2% males and 47.8% females). The proportion in each BMI categorization changed significantly from 2009 to 2016 in all groups (*p* < 0.001). Increases in the Overweight and Moderate or Severe Obesity categories were observed in all age groups (2–5, 6–10, and 2–10), and increases in Obese were observed in 6–10-year olds. Most shifts occurred from healthy-weight individuals increasing in BMI category.

***Conclusions:*** We observed a significant increase in the proportion of children with overweight and obesity in a nationally representative MHS cohort from 2009 to 2016. The prevalence of obesity, but not overweight, in our cohort mirrored the civilian population. Increasingly heavier MHS and civilian children have implications for our future military force, as they are ineligible for military service if unable to meet weight standards.

## Introduction

According to the CDC National Center for Health Statistics,^1^ obesity prevalence across different age groups within the pediatric population is increasing.^2^ Hales et al. noted increasing obesity prevalence in 2–5-year olds, 6–11-year olds, and 12–19-year olds from the mid-1960s until the mid-2010s. From the same group, other Hales et al. found a similar trend, noting that obesity prevalence in 2–19-year-old youth has increased from 13.9% in 1999–2000 to 18.5% in 2015–2016.^3^ While weight issues are multifactorial in nature, the increasing trend in youth with overweight and obesity over the past decades is clear.

Ultimately, children and adolescents grow into adults and provide the pipeline for future generations of military service members. With a 2017 simulation study, Ward et al. projected that given our current childhood obesity prevalence, about 57% of children would develop obesity by age 35 years.^4^ As it currently stands, about 27% of young civilian adults from 17 to 24 years of age are ineligible to serve in the military due to their inability to meet weight standards.^5^

In this study, we sought to better understand the changes in BMI categorizations in a Military Health System (MHS) pediatric cohort who are a universally insured segment of the United States (US) population. We hypothesize that the MHS pediatric cohort mirrors the changes seen in civilian and non-MHS counterparts, ultimately furthering the negative impact of increasing pediatric obesity on the strength of our future military fighting force.

## Materials and Methods

### Study Design and Data Source

Utilizing the Military Health System Data Repository (MDR), we conducted a retrospective study on a cohort of pediatric patients 2–10 years of age during fiscal year (FY) 2009. We then identified the same cohort in FY 2016 and recorded their age, gender, and height and weight measurements. The MDR houses all encounter and health care claims data for TRICARE beneficiaries receiving care at both military treatment facilities (direct care) and at civilian fee-for-service facilities (purchased care). TRICARE is the Department of Defense health care insurance product that covers ∼9.6 million beneficiaries, which consists of Active Duty military personnel, retirees, and their dependents.^6^ Due to the unavailability of height and weight measurements in purchased care claims data, only encounter data from pediatric patients in the direct care system were used in this study.

### Study Population and Analyses

We identified an initial cohort of 132,872 pediatric patients who were 2–10 years old in FY 2009 and whom we were able to track in FY 2016. To calculate BMI, the identified pediatric cohort was required to have complete data for age, gender, height, and weight in both FY 2009 and 2016 (cohort age will be 9–17 years in 2016). We then applied the CDC Pediatric BMI *z*-score and percentile calculations to the cohort using the CDC SAS program.^7^ At the suggestion of the CDC, and to reduce bias in *z*-score calculations, 6 months or 0.5 years was added to cohort ages. The *z*-score program also identifies and flags extreme values as biologically implausible, with a BMI *z*-score <−5 as the lower limit and a BMI *z*-score >6 as the upper limit.^7^ Pediatric patients with extreme BMI values in either FY 2009 or 2016 were treated as outliers and were excluded from analyses.

BMI categories were classified based on calculated percentile for age and gender, with the CDC advising to categorize obese and moderate or severe obesity categories relative to the 95th percentile. The pediatric BMI category percentile ranges are as follows: underweight, less than the 5th percentile; healthy weight, 5th percentile to less than the 85th percentile; overweight, 85th to less than the 95th percentile; obese, 95th to less than the 120th percentile; moderately obese, 120th to less than the 140th percentile; and severely obese, 140th percentile or greater.^1^

Descriptive statistics were performed on patient demographics such as gender and sponsor's rank and service, stratified by age groups of interest (2–5, 6–10, and 2–10 year olds). We also calculated the percent change in BMI categories from FY 2009 to 2016 and utilized the Stuart-Maxwell test of marginal homogeneity to determine if there were statistical differences in the reported BMI distributions. The Stuart-Maxwell test was chosen because it is appropriate for the paired design of the study, in which each subject's BMI status was assessed at two different time points. Statistical significance was indicated by *p* < 0.001. Finally, the overall prevalence of BMI categories in FY 2009 compared to FY 2016 was evaluated. All statistical analyses were performed using SAS version 9.4 and STATA version 16. This study was reviewed and exempted by the Uniformed Services University Institutional Review Board.

## Results

We identified a total of 130,675 children eligible for inclusion in this cohort study. Complete patient demographics are noted in Table 1. We broke down the information into FY 2009 2–5, 6–10, and 2–10 years old groupings, and we identified gender, military sponsor's rank, and military sponsor's service. About 48% were female and 52% were male. About 70% of the military sponsors were enlisted, 25% were officers, and 3% were warrant officers. About 45% of the sponsors were Army, 20% were Navy, 28% were Air Force, and 6% were Marines.

**Table 1.** Cohort Demographics in Fiscal Year 2009, *n* = 130,675

	*n* (% of age group)		
	2–5 years old (*n* = 66,170)	6–10 years old (*n* = 64,505)	2–10 years old (*n* = 130,675)
Gender			
Male	34,919 (52.8)	33,258 (51.6)	68,177 (52.2)
Female	31,251 (47.2)	31,247 (48.4)	62,498 (47.8)
Sponsor's rank			
Enlisted	47,898 (72.4)	44,925 (69.7)	92,823 (71.0)
Officer	16,695 (25.2)	17,330 (26.9)	34,025 (26.0)
Warrant officer	1474 (2.2)	2087 (3.2)	3561 (2.7)
Missing	103 (0.2)	163 (0.3)	266 (0.2)
Sponsor's service			
Army	28,881 (43.7)	29,787 (46.2)	58,668 (45.0)
Navy	13,479 (20.4)	12,521 (19.4)	26,000 (20.0)
Air force	18,669 (28.2)	17,864 (27.7)	36,533 (28.0)
Marines	4240 (6.4)	3539 (5.5)	7779 (6.0)
Coast guard	676 (1.0)	615 (1.0)	1291 (1.0)
Other/missing	225 (0.3)	179 (0.3)	404 (0.3)

For Tables 2–4, we looked at the percent change in BMI categories for 2–5, 6–10, and 2–10-year-old patients in FY 2009 compared to FY 2016. In this study, we used the Stuart-Maxwell test of marginal homogeneity. All three age group comparisons resulted in a *p* < 0.001, signifying that there is a statistically significant difference in weight categories between 2009 and 2016.

**Table 2.** Change in BMI Category Distributions in Fiscal Year 2009 vs. FY 2016 for the Total Pediatric Cohort (2–10-Year Olds in FY 2009), *n* = 130,675^a^

FY 2009 BMI categories for total cohort	FY 2016 BMI categories				
	% of row total				
	Underweight (%)	Healthy weight (%)	Overweight (%)	Obese (%)	Moderately or severely obese (%)
Underweight (*n* = 6768)	24.2	68.8	3.5	3.0	0.5
Healthy weight (*n* = 93,164)	3.7	77.9	9.5	8.1	0.9
Overweight (*n* = 7955)	0.4	46.2	23.2	27.1	3.2
Obese (*n* = 20,398)	0.4	34.0	17.3	36.8	11.6
Moderately or severely obese (*n* = 2390)	0.5	13.3	6.5	30.0	49.7

^a^ *p* < 0.001 for above table based on Stuart-Maxwell test of marginal homogeneity.

FY, fiscal year.

For Table 2, we looked at the percent change in 2–10 years old cohort BMI categories in FY 2009 vs. FY 2016. For orientation purposes, the left-most column provides the BMI category for FY 2009 and the remaining columns provide the percentage BMI category for FY 2016. As an example, the FY 2009 *Healthy Weight* BMI category has *n* = 93,164. For FY 2009, about 4% reclassify as *Underweight*, 78% as *Healthy Weight*, 10% as *Overweight*, 8% as *Obese*, and 1% as *Moderately or Severely Obese* (*MSOb*) in FY 2016. Approximately 20% of whom were *Healthy Weight* in FY 2009 were reclassified as *Overweight*, *Obese*, or *MSOb*; ∼30% of whom were *Overweight* in FY 2009 were reclassified as *Obese* or *MSOb*; ∼12% of whom were *Obese* in FY 2009 were reclassified as *MSOb*; and ∼50% of FY 2009 *MSOb* remained there. The biggest increase from 2009 vs. 2016 was in the *Overweight* group, which went from *n* = 7955 (FY 2009) to *n* = 14,568 (FY 2016).

For Table 3, we looked at the percent change in 2–5 years old cohort BMI categories in FY 2009 vs. FY 2016. In this study, we combined *Obese* and *MSOb* since the number in *MSOb* was low. Approximately 18% of 2–5-year-old *Overweight* remained such and 26% increased to *Obese* categories. Approximately 40% of 2–5 year olds with obesity remained in that classification as well. The largest increase from 2009 vs. 2016 was in the *Overweight* group, which went from *n* = 2103 (FY 2009) to *n* = 6972 (FY 2016).

**Table 3.** Change in BMI Category Distributions in Fiscal Year 2009 vs. FY 2016 for Pediatric Cohort Patients 2–5 Years of Age, *n* = 66,170^a^

FY 2009 BMI categories for cohort patients 2–5 years of age	FY 2016 BMI categories			
	% of row total			
	Underweight (%)	Healthy weight (%)	Overweight (%)	Obese (%)
Underweight (*n* = 4092)	22.9	68.3	4.3	4.4
Healthy weight (*n* = 48,123)	4.4	75.6	9.4	10.6
Overweight (*n* = 2013)	0.7	55.0	18.2	26.1
Obese (*n* = 11,942)	0.6	43.3	15.9	40.2

^a^ *p* < 0.001 for above table based on Stuart-Maxwell test of marginal homogeneity.

For Table 4, we looked at the percent change in 6–10 years old cohort BMI categories in FY 2009 vs. FY 2016. In this study, we combined *Obese* and *MSOb* since the number in *MSOb* was low. Approximately 25% of 6–10 year olds with *Overweight* remained such and 32% increased to *Obese* categories. Approximately 64% of 6–10 year olds with Obesity remained in that classification. The largest increases from 2009 vs. 2016 were in the *Overweight* and *Obese* groups.

**Table 4.** Change in BMI Category Distributions in Fiscal Year 2009 vs. FY 2016 for Pediatric Cohort Patients 6–10 Years of Age, *n* = 64,505^a^

FY 2009 BMI categories 6–10 years old (n = 64,505)	FY 2016 BMI categories			
	% of row total			
	Underweight (%)	Healthy weight (%)	Overweight (%)	Obese (%)
Underweight (*n* = 2676)	26.1	69.5	2.3	2.1
Healthy weight (*n* = 45,041)	2.9	80.3	9.5	7.2
Overweight (*n* = 5942)	0.2	43.2	24.9	31.8
Obese (*n* = 10,846)	0.2	19.1	16.4	64.3

^a^ *p* < 0.001 for above table based on Stuart-Maxwell test of marginal homogeneity.

**Figure 1.**
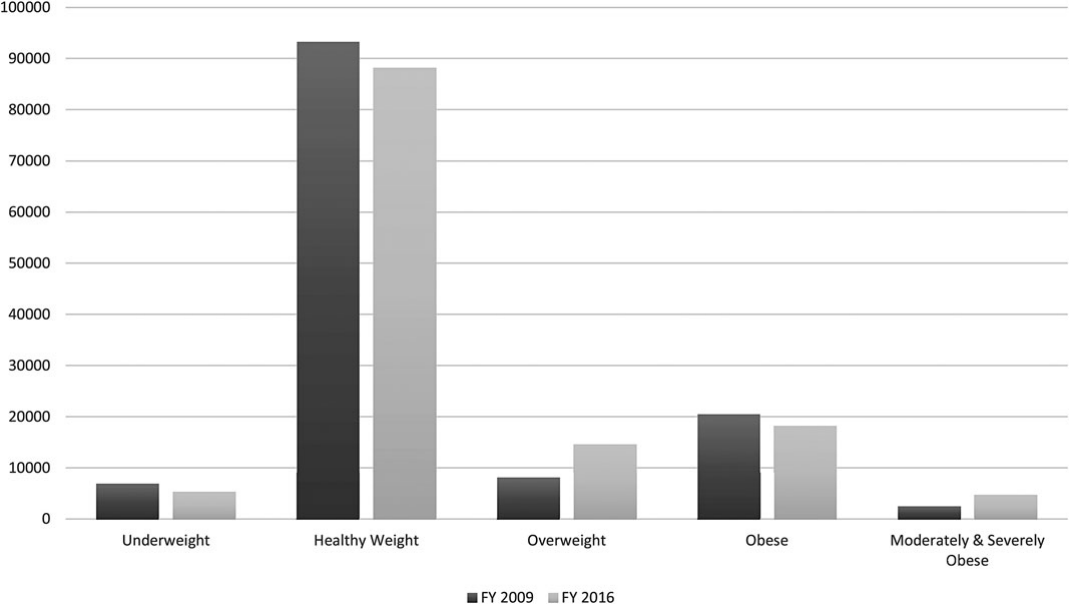
Prevalence distributions of BMI categories in FY 2009 compared to FY 2016 for the total cohort. Cohort ages are 2–10 years old in FY 2009 and 9–17 years old in FY 2016. *n* = 130,675. FY, fiscal year.

**Figure 2.**
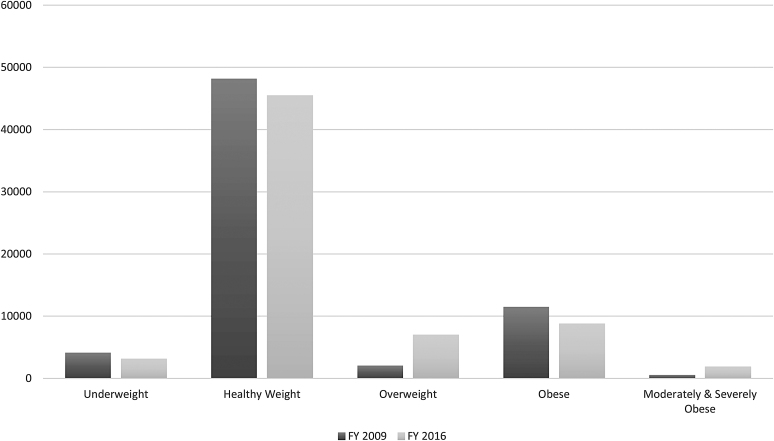
Prevalence distributions of BMI categories in FY 2009 compared to FY 2016 for pediatric cohort patients 2–5 years of age in FY 2009. Cohort ages in FY 2016 are 9–12 years old. *n* = 66,170.

**Figure 3.**
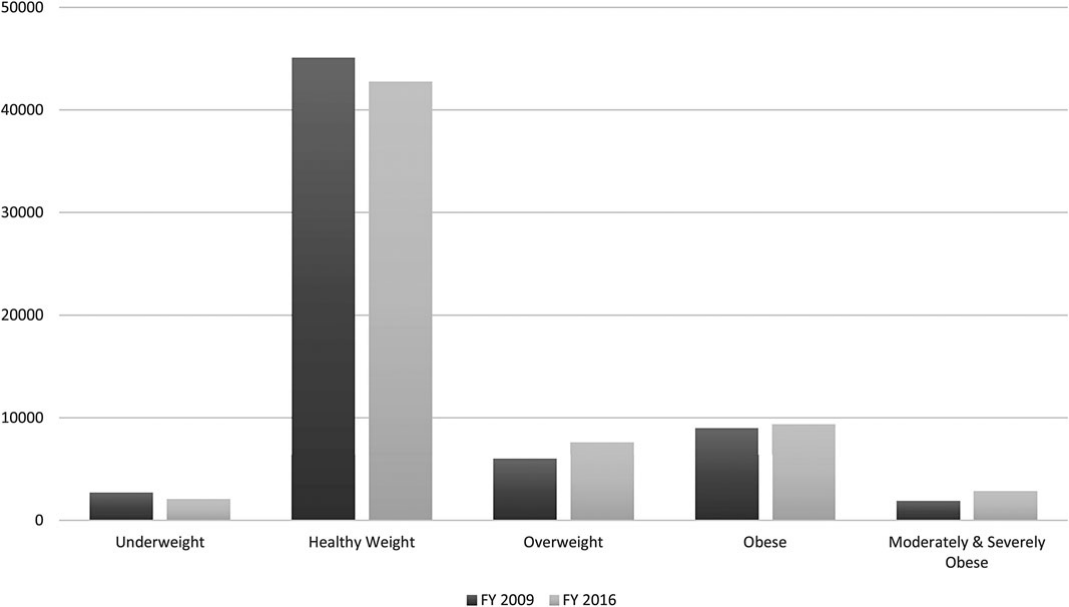
Prevalence distributions of BMI categories in FY 2009 compared to FY 2016 for pediatric cohort patients 6–10 years of age in FY 2009. Cohort ages in FY 2016 are 13–17 years old. *n* = 64,505.

We also examined the overall prevalence of the BMI categories for 2–10, 2–5, and 6–10 years old cohorts, respectively. Figures 1–3 provide a visual representation of the observed changes; however, the exact proportions will be reported and discussed. For the 2–10 years old FY 2009 cohort, the prevalence of the *Overweight* category increased from 6.1% to 11.2%, while the *MSOb* category increased twofold from 1.8% to 3.6%. For the 2–5 years old FY 2009 cohort, the prevalence of the *Overweight* category increased threefold from 3.0% to 10.5%. For the 6–10 years old FY 2009 cohort, the prevalence of the *Overweight* category increased from 9.2% to 11.8%, while the *Obese* category increased from 16.8% to 18.9%.

## Discussion

In the overall US population, overweight and obesity rates in pediatric patients are increasing.^2^ In this study, we evaluated an MHS pediatric cohort to see if the trends are similar. In our group for 2–10-year olds, there was 11.2% *Overweight* categorization and 17.5% *Obese* plus *MSOb* categorization. Interestingly, the cohort's obesity (but not overweight) prevalence mirrors the civilian population. Then, our *Overweight* grouping was at about 14% and *Obese* was at about 10%. While these rates differ, the grouping changes are of most clinical relevance.

For 2–5-, 6–10-, and 2–10-years old individuals, the biggest shift seemed to be from a *Healthy Weight* categorization to an *Overweight* one as discussed in the Results section above. This conclusion is based on percentages and total number as indicated in Tables 2–4. As clinicians, it was most interesting that 18.2% of 2–5 year olds with *Overweight* remained such, and with 26.1% increasing to the *Obese* categorization. Of the *Obese* category, 40.2% remained such from 2009 to 2016. Overall, this may indicate that children do not necessarily grow out of their weight issues, and despite being young, they should be a focus of clinical intervention focusing on weight maintenance.^4^

Theses shifts are important in terms of health outcomes. Pediatric overweight and obesity status are associated with numerous health issues, to include a multitude of metabolic, cardiovascular, pulmonary, musculoskeletal, gastrointestinal, and renal disorders.^8^ Also, there has been a noted association with premature mortality in adulthood^8^ and significant economic impacts over time. In a 2009 study, Trasande and Chatterjee found an association between elevated childhood BMI and a $14.1 billion cost increase in prescriptions, emergency room visits, and outpatient clinic visits each year from 2002 to 2005; and $237.6 million for obesity-associated hospitalizations in 2005.^9^ In 2014, it was estimated that a 10-year-old child with obesity would incur $19,000 more in direct medical costs over a lifetime compared to a 10-year-old healthy-weight child.^10^ In a 2015 study, Brookings Institute researchers estimated that the lifetime societal costs were about $92,000 greater for a person with obesity ($2013), and societal costs could exceed $1.1 trillion for the more than 12 million youth with obesity, who become adults with obesity.^11^

This economic burden from obesity also falls on the ranks within active duty military and new recruits. In a 2007 study of 18-year-old US military recruits, Hsu et al. found that the prevalence of *Overweight* (defined as BMI 25–<30 kg/m^2^) increased from 22.8% in 1993 to 27.1% in 2006, and the prevalence of *Obesity* (defined as BMI ≥30 kg/m^2^) increased from 2.8% in 1993 to 6.8% in 2006.^12^ Similar trends were found by Hruby et al. when examining overweight and obesity in new US Army recruits of all ages from 1989 to 2012. The prevalence of *Overweight* generally increased from 25.8% in 1989 to 37.2% in 2012, and the prevalence of *Obesity* increased from 5.6% in 1989 to 8.0% in 2012.^13^ These studies confirm that the US military is recruiting from a population with obesity.

In our current military, the Department of Defense spends over $1.1 billion in annual costs to help active-duty service members maintain their weight standards, while also covering weight-related health costs and lost work productivity.^5^ For dependents of active-duty service members, the Department of Defense spends over $1 billion for weight-related comorbidities.^5^ The health and socioeconomic costs of increasing prevalence of overweight and obesity in our youth and adolescents are clear, and that burden only increases as they enter either the civilian or military workforce. Ultimately, investment in promoting health and wellness in children and adolescents, such as bringing home healthy lifestyle habits learned from school-based intervention programs,^14^ would result in a healthier fighting force and in an improved national security, both today and tomorrow.^15^

The main strength of this study was the large population of 130,675 children. Also, the 7-year follow-up period between 2009 and 2016 provided time for development in the cohort. Limitations resulted from the fact that the BMI calculation came off of the first data log for height and weight in the FY and out of administrative data.

For next steps, we would consider a breakdown of the cohort based on racial and ethnic information, as certain minority groups have higher rates of obesity compared to nonminority groups.^1^ Additional research might focus on the impact of duty station/geographic location throughout the US and if MHS beneficiaries mirror the overweight and obesity prevalence of that specific state.

## Conclusions

In conclusion, we evaluated a cohort of 2–10-year-old pediatric patients in 2009 compared to the same group in 2016. We identified an increased prevalence in *Overweight* and *MSOb* categorizations for FY 2009 2–10-year olds, *Overweight* for FY 2009 2–5-year olds, and *Overweight* and *Obese* for FY 2009 6–10-year olds. Further research is needed to understand the age, gender, socioeconomic status, and racial and ethnic breakdowns of these differences and their interactions. Future research of this nature could provide insight into the necessity of targeted interventions, as well as where they will have the most impact. Overall, although an increasingly heavier MHS (and civilian) pediatric population has implications for our future fighting force, those who cannot meet weight standards for military service are ineligible to serve our country.
